# Overexpression of *CBS* and *CSE* genes affects lifespan, stress resistance and locomotor activity in *Drosophila melanogaster*

**DOI:** 10.18632/aging.101630

**Published:** 2018-11-08

**Authors:** Mikhail Shaposhnikov, Ekaterina Proshkina, Lyubov Koval, Nadezhda Zemskaya, Alex Zhavoronkov, Alexey Moskalev

**Affiliations:** 1 Engelhardt Institute of Molecular Biology, Russian Academy of Sciences, 119991 Moscow, Russia; 2 Institute of Biology of Komi Science Center of Ural Branch of RAS, 167982 Syktyvkar, Russia; 3 Insilico Medicine, Inc, Johns Hopkins University, Baltimore, MD 21218, USA; 4 Moscow Institute of Physics and Technology, Dolgoprudny 141700, Russia

**Keywords:** *Drosophila*, hydrogen sulfide, lifespan, locomotor activity, paraquat, hyperthermia, desiccation, starvation

## Abstract

Recent experimental studies highlighted the role of hydrogen sulfide (H_2_S) in aging and longevity. The cystathionine ß-synthase (CBS) and cystathionine γ-lyase (CSE) are the key enzymes responsible for H_2_S production. Here we investigated the geroprotective effects of *CSE* and *CBS* overexpression in *Drosophila*. Overexpression of *CSE* did not affect a lifespan and decrease (mitochondrial form of *CSE*) or increase (cytoplasmic form of *CSE*) age dynamics of locomotor activity, while overexpression of *CBS* increase median (by 12.5%) and maximum (by 6.9%) lifespan and locomotor activity. Increasing of both *CSE* and *CBS* expression levels resulted in thermotolerance, but the resistance to combination of arid and food-free conditions decreased. The resistance to oxidative stress (paraquat) was not affected in flies with overexpression of *CBS* and cytoplasmic *CSE*, but decreased in flies overexpressing mitochondrial form of *CSE*. Thus, transgene overexpression of the *CSE* and *CBS* in *Drosophila* induce similar effects on stress-resistance and locomotor activity, however lifespan extending effect was revealed for *CBS* overexpression only.

## Introduction

Aging is a multifactorial process characterized by a widespread loss of homeostasis, leading to gradual decrease in functional capacity at all levels of biological organization which, reduced resistance to environmental stresses and exponential increase in probability of death [1]. Evolutionarily conserved stress- and nutrient-responsive signaling pathways (such as growth hormone/IGF-1, NAD^+^/sirtuins, PI3K/AKT/mTOR, AMPK/mTOR and JNK) regulate homeostasis and longevity by coordinating metabolic functions, cell growth and proliferation, and stress responses throughout the organism [1,2]. A number of genetic, pharmacological or environmental interventions in activities of these pathways are known to induce beneficial pleiotropic effects on cell and extend lifespan in model organisms [3].

Recent experimental studies highlighted a potential role of hydrogen sulfide (H_2_S) in the regulation of lifespan and aging. Endogenous H_2_S is the product of metabolism of sulphur containing amino acids, methionine and cysteine in evolutionarily conserved transsulfuration pathway (TSP) [4,5]. The TSP is activated in response to such anti-aging intervention as dietary restriction (DR), and H_2_S apparently mediates the pleiotropic DR benefits including longevity and stress resistance in different models, from yeast, worms and flies to mice [4,5]. H_2_S affects aging by sulfhydration of target proteins and influencing cellular signaling pathways [6]. H_2_S influences aging-related processes such as cellular bioenergetics, autophagy, inflammation, oxidative stress, proliferation and differentiation of stem cell, cellular senescence, cell death and cellular metabolism [6–10].

In animals and particular in *Drosophila melanogaster*, H_2_S is produced by two key enzymes of the TSP, cystathionine β-synthase (CBS) and cystathionine γ-lyase (CSE) [4,11]. The lifespan extension under DR in *Drosophila* was associated with increased CBS activity [4]. Overexpression of *CBS* homologs in nematodes and flies increased lifespan independent of diet, while RNAi-mediated knocking down of the same genes or inhibition of the CSE in *Drosophila* using propargylglycine limited or abrogated DR-mediated lifespan extension [4,12]. Mice with loss-of-function mutations in *CBS* and *CSE* genes demonstrated increased plasma homocysteine level, growth retardation and significantly reduced lifespan (about 5 and 12 weeks, respectively) [13,14]. Exposing to H_2_S-containing atmosphere has been shown to induce thermotolerance and increase longevity in *Caenorhabditis elegans* [15]. Chronic treatment of *C. elegans* with an exogenous H_2_S (via the slow-releasing donor GYY4137) extended median survival and increased tolerance towards oxidative and endoplasmic reticulum stress [16]. Thus, both exogenous and endogenous H_2_S are important for stress resistance, longevity and health of the model organisms. However, effects of the overexpression of the genes controlling endogenous H_2_S synthesis on the stress resistance, lifespan and locomotor activity are not investigated fully. In the present work, we for the first time investigated whether ubiquitous transgenic overexpression of *CSE* (coding mitochondria- and cytoplasm-located forms of CSE) affect the resistance to various stress factors (oxidative stress, hyperthermia and combination of arid and food-free conditions), longevity and locomotor activity in *Drosophila* *melanogaster* and compared obtained results with the effects of pro-longevity gene *CBS* overexpression.

## RESULTS

### Overexpression of transgenes

Using FlyBase search [17] we identified 3 genes responsible for H_2_S production in *Drosophila*, *CG12264* coding for mitochondria-located enzyme with CSE activity [18], *CG5345* that codes the cytoplasm-located orthologue of *CSE* [11] and *CG1753* that is the single homolog of *CBS* [4]. Using cDNA of these genes 3 UAS lines were generated (Table S1).

In their previous report, Kabil [4] showed that ubiquitous (driven by *tubulin-GAL4*) or neuron-specific (driven by *Elav-GAL4*) transgenic overexpression of *CBS* enhanced *Drosophila* longevity, while *CBS* overexpression in abdominal fat body (driven by GeneSwitch S1-106) and in gut (driven by GeneSwitch TIGS-2) had no effect on lifespan [4]. To further study the role of genes affecting H_2_S production in longevity of *Drosophila*, we tested whether ubiquitous overexpression of *CBS* and *CSE* under the control of *da-GAL4* driver also increase resistance to stress factors, prolong lifespan and improve locomotor performance.

The expression levels of transgenes were examined by qRT-PCR assays before further analysis (Figure S1). We found 2.4-, 6.1- and 1.9-fold induction (p<0.05) of *CSE[LD22661]*, *CSE[LD22255]*, *CBS[LD21426]*, respectively compared with their isogenic UAS controls (Figure S1, Table S3). The level of transgene expression was found to depend on the UAS transgene.

### Lifespan

**Figure 1:**
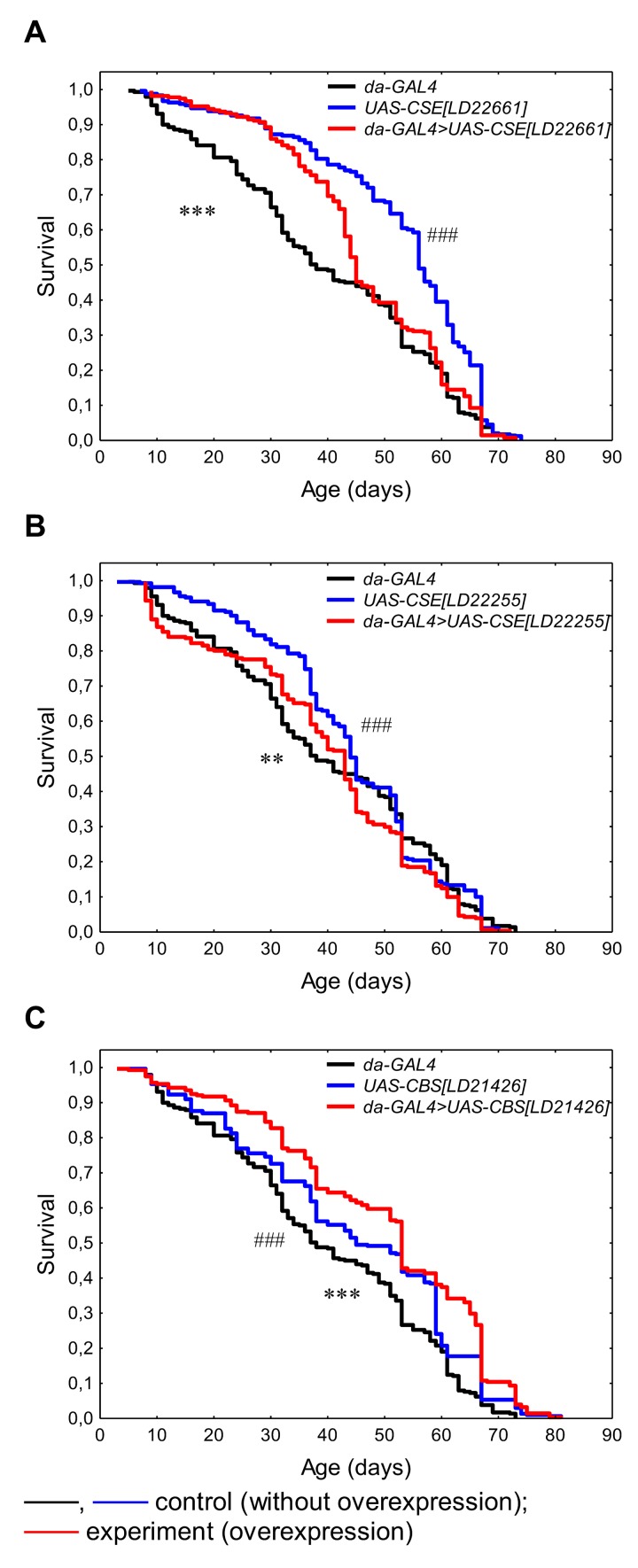
**The effects of constitutive ubiquitous overexpression of *CSE[LD22661]* (A), *CSE[LD22255]* (B), *CBS[LD21426]* (C) on lifespan.** *p<0.05, **p<0.01, ***p<0.001, *da-GAL4>UAS* vs *da-GAL4*; ^#^p<0.05, ^##^p<0.01, ^###^p<0.001, *da-GAL4>UAS* vs *UAS*, Kolmogorov-Smirnov test.

The activation of constitutive *CSE[LD22661]* overexpression leaded to prolongation by 15.6% relative to *da-GAL4* parental control line (p<0.05). At the same time, we observed the decline of median and maximum lifespan in males with *CSE[LD22661]* overexpression compared with maternal control line *UAS-CSE[LD22661]* by 24.4% (p<0.0001) and 3.1% (p<0.01), respectively (Table 1, Figure 1A). The increase in median lifespan by 11.6% (p<0.05) was caused by upregulation of *CSE[LD22255]* relative to *da-GAL4* parental control line, but the lifespan differences in comparison with the *UAS-CSE[LD22255]* maternal control line were not statistically significant (Table 1, Figure 1B). Thus, overexpression of both forms of the *CSE* gene did not lead to an increase in lifespan relative to the both paternal and maternal control lines. As we know the effect of *CSE* overexpression on the longevity was not previously studied. As it was established by Kabil [4] pharmacological inhibition of the CSE using propargylglycine reversed lifespan extension induced by DR, and there was no effect of CSE inhibition on fully fed flies [4]. Overexpression of *CSE* in *Drosophila* SCA3-model restored protein persulfidation, decreased oxidative stress, dampened the immune response and improved SCA3-associated tissue damage and neurodegeneration [19]. Despite these published beneficial effects on aging-related processes, we did not observe the positive effect of *CSE* overexpression on the lifespan.

**Table 1:** The effects of *CSE* and *CBS* overexpression on lifespan.

Genotype	Variant	M (days)	dM (%)	Log-Rank Test (p)	90% (days)	d90% (%)	Wang-Allison Test (p)	MRDT (days)	dMRDT (%)	n
*da-GAL4>UAS-CSE[LD22661]*	overexpression	45			65			9		270
*da-GAL4*	control (parental)	38	-15.6	p<0.05 (0.0305)	63	-3.1	p>0.05 (0.051)	13.6	51.1	289
*UAS-CSE[LD22661]*	control (maternal)	56	24.4	p<0.0001 (4.59e-08)	67	3.1	p<0.01 (0.009)	7.5	-16.7	243
*da-GAL4>UAS-CSE[LD22255]*	overexpression	43			61			12		281
*da-GAL4*	control (parental)	38	-11.6	p<0.05 (0.0305)	63	3.3	p>0.05 (0.051)	13.6	13.3	289
*UAS-CSE[LD22255]*	control (maternal)	44	2.3	p>0.05 (0.196)	67	9.8	p>0.05 (0.799)	9.6	-20	270
*da-GAL4>UAS-CBS[LD21426]*	overexpression	53			72			11.2		278
*da-GAL4*	control (parental)	38	-28.3	p<0.05 (0.0305)	63	-12.5	p>0.05 (0.051)	13.6	21.4	289
*UAS-CBS[LD21426]*	control (maternal)	45	-15.1	p>0.05 (0.0707)	67	-6.9	p<0.05 (0.013)	13.1	17	299

M - median lifespan; 90% - age of 90% mortality (maximum lifespan); MRDT - mortality rate doubling time; dM, d90%, dMRDT - differences between median lifespan, age of 90% mortality, and MRDT of control and experimental flies, respectively; n - number of flies.

The *CBS[LD21426]* overexpression increased median (by 15.1-28.3%, p<0.05) and maximum (by 6.9-12.5%, p<0.05) lifespan relative to *UAS-CBS[LD21426]* maternal and *da-GAL4* parental control lines, respectively (Table 1, Figure 1C). In addition, *CBS[LD21426]* overexpression decreased MRDT (by 17% and 21.4%, respectively) suggesting that geroprotective effect of CBS in associated with prolongation of healthspan. The observed lifespan extending effect of *CBS* ubiquitous overexpression is consistent with the results of Kabil [4].

### Stress resistance

**Figure 2:**
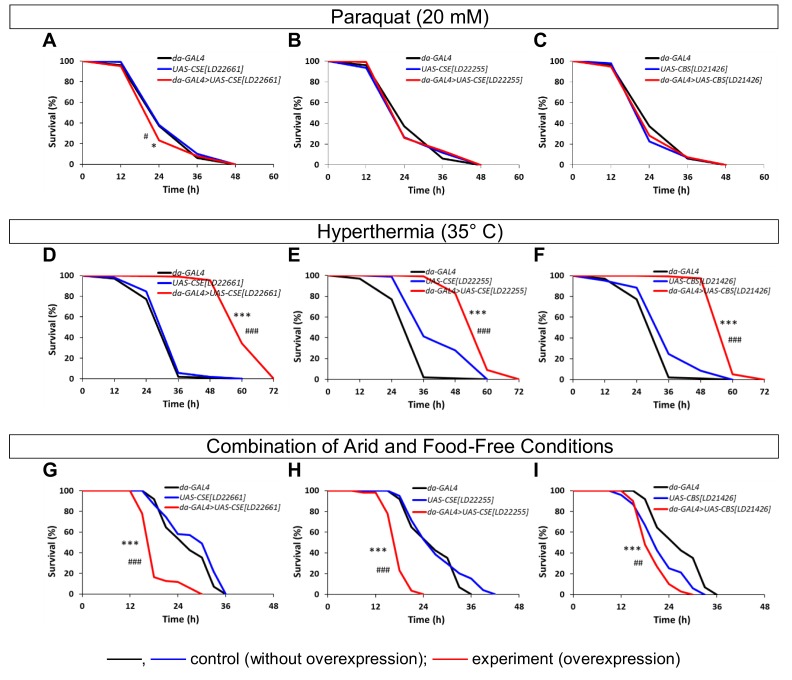
**The effects of constitutive ubiquitous overexpression of *CSE* and *CBS* genes on resistance to stress factors (paraquat (A-C), hyperthermia (D-F) and combination of arid and food-free conditions (G-I)).** *p<0.05, **p<0.01, ***p<0.001, Fisher's exact test (*da-GAL4>UAS* vs *da-GAL4*); #p<0.05, ##p<0.01, ###p<0.001, Fisher's exact test (*da-GAL4>UAS* vs *UAS*).

We also analyzed the resistance to different kinds of stress factors of flies with overexpression of the *CBS* and *CSE* genes and the control lines. The survival curves and times of 25%, 50%, 75% and 90% mortality in flies under conditions of paraquat (oxidative stress), hyperthermia, and combination of arid and food-free conditions were estimated (Table S4, Figure 2). The *CSE[LD22661]* overexpression leaded to marginal decrease (p<0.05) of resistance to paraquat (Figure 2A), while *CSE[LD22255]* and *CBS[LD21426]* overexpression had no effect (p>0.05) on resistance to oxidative stress (Figures 2B and C). According to published data exogenous and endogenous H_2_S has been shown to improve the capacity of model animals to survive in conditions of oxidative stress. Pharmacological H_2_S donor (GYY4137) protected wild-type *C. elegans* against paraquat poisoning [20]. In *D. melanogaster* the X-ray radiation-induced embryonic mortality were found to be reduced by H_2_S treatment [21]. Our data demonstrated that overexpression of the *CBS* and *CSE* is not sufficient to increase tolerance to oxidative stress in *Drosophila*.

We also found that *CSE[LD22661]*, *CSE[LD22255]* and *CBS[LD21426]* upregulation provoked significant elevation of resistance to hyperthermia (Figures 2D-F). The obtained results are consistent with the published effects of exogenous H_2_S. Exposure of *C. elegans* increased concentrations of H_2_S has been shown to enhance thermotolerance [15].

The significant decrease of resistance to combination of arid and food-free conditions was observed (p<0.001, Figures 2G-I). As known from the published data exposure to H_2_S emanated from a K_2_S donor significantly increased survival of flies under arid and food-free conditions, but not under humid and food-free conditions, demonstrating that H_2_S plays a role in desiccation tolerance but not in nutritional stress alleviation [22]. On the other hand, overexpression of the *CBS*, was sufficient to promote fat deposition in flies [4]. It is well known that *Drosophila* are better able to resist starvation by sequestering more body lipid reserves [23]. Thus the final role of endogenous H_2_S in the formation of resistance to starvation and desiccation has to be clarified.

### Locomotor activity

**Figure 3:**
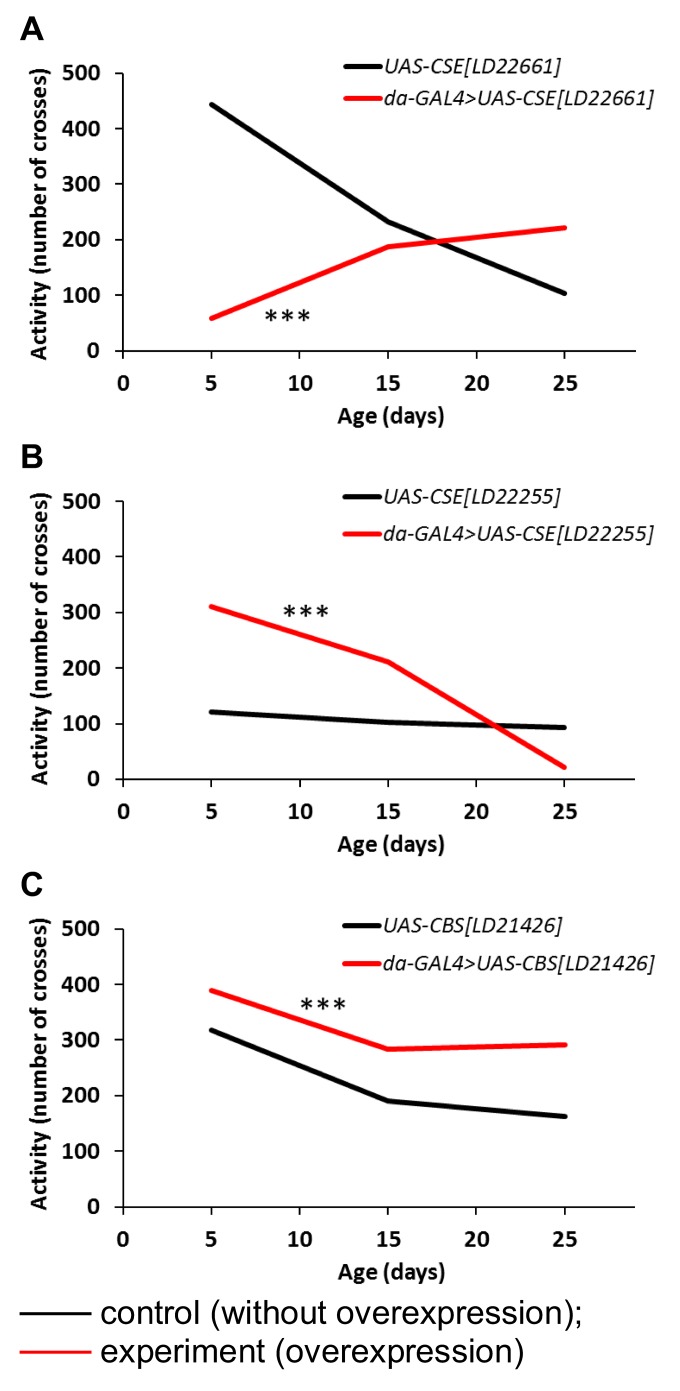
**The effects of constitutive ubiquitous overexpression of *CSE[LD22661]* (A), *CSE[LD22255]* (B), *CBS[LD21426]* (C) on age-dependent changes in spontaneous locomotor activity.** Locomotor activity was defined as averaged number of sensor crosses during 3 min by 30 flies. *р<0.05, **р<0.01, ***р<0.001, χ^2^.

The influence of *CBS* and *CSE* overexpression on locomotor activity was estimated in flies at the age of 5, 15 and 25 days (Figure 3). It was found that *UAS-CSE[LD22661]* overexpression induced decline of spontaneous locomotor activity in the first part of life and increase of this parameter in the second part of male’s life (Figure 3A). In *UAS-CSE[LD22255]* overexpressing males locomotor activity is higher than in control group in the first and second third of life but it is lower in the last one. (Figure 3B). The *UAS-CBS[LD21426]* overexpressing males were characterized by high longlife locomotor activity (Figure 3C).

## DISCUSSION

Therefore, we analyzed the effects of constitutive ubiquitous overexpression of *CSE[LD22661]*, *CSE[LD22255]* and *CBS[LD21426]* on the *Drosophila melanogaster* lifespan, stress resistance (paraquat (oxidative stress), hyperthermia, and combination of arid and food-free conditions) and healthspan (locomotor activity). We showed that the overexpression of cystathionine β-synthase gene (*CBS[LD21426]*) was characterized by the highest geroprotective efficiency. The *CBS[LD21426]* upregulation causes increase of median lifespan (by 12.5%) and maximum lifespan (by 6.9%) in males (p<0.05) relative to parental lines. At the same time the overexpression of this gene is resulted in increased thermotolerance (p<0.05) and locomotor activity (p<0.001), but decline resistance to arid and food-free conditions (p<0.001). The overexpression of cystathionine γ-lyase genes (*CSE[LD22661]* and *CSE[LD22255]*) did not lead to geroprotective effects and caused decline of resistance to arid and food-free conditions (p<0.001), but simultaneously, the elevation of resistance to hyperthermia was observed (p<0.001). The differences in the effects of *CBS* and *CSE* genes overexpression on the lifespan can be partly explained by different roles of the enzymes encoded by these genes in the TSP. While the CBS catalyzes the first and rate-determining step in the TSP, which involves the pyridoxal 5′-phosphate-dependent condensation of serine and homocysteine to form cystathionine and H_2_S, the CSE uses cystathionine as a substrate for cysteine and H_2_S production [4]. Thus, CBS can partially limit the effects of CSE overexpression on H_2_S production and lifespan.

We showed that *CBS* and *CSE* overexpression induce elevated resistance to hyperthermia. And in case of *CBS* overexpression thermotolerance is accompanied by an increase in lifespan. The common role of H_2_S in thermotolerance and longevity was revealed in publication of Miller and Roth [15]. They showed that elevated resistance to hyperthermia and increased lifespan in *C. elegans* after exposure to H_2_S require activity of silent information regulator 2 (SIR2) but are independent of the insulin signaling pathway, mitochondrial dysfunction, and caloric restriction [15]. Our results confirm their assumption that sirtuin-dependent mechanisms by which H_2_S increases thermotolerance and lifespan in nematodes may be evolutionary conserved. In addition the overexpression of *dSir2* extended lifespan in different model organisms including *Saccharomyces cerevisiae* [24], *C. elegans* [25], *D. melanogaster* [26] and *Mus musculus* males [27]. We also found that overexpression of *CBS[LD21426]* and *CSE[LD22255]* is associated with the increase of locomotor activity. This effect can be explained by stimulation of cellular bioenergetics with H_2_S produced by CSE and CBS enzymes [9].

**Figure 4:**
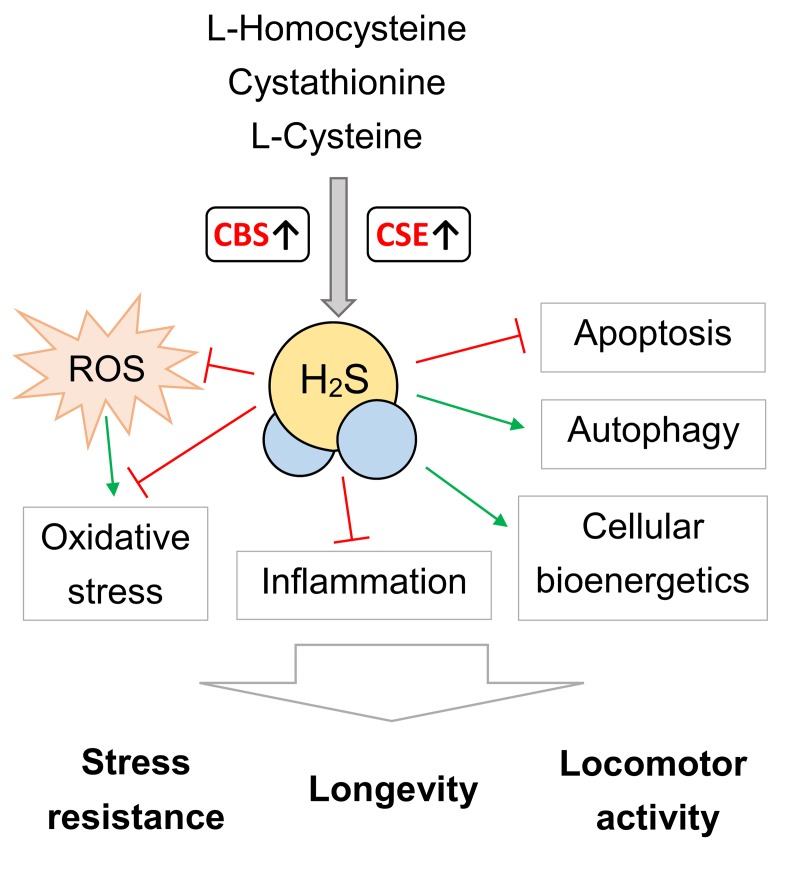
**The possible mechanisms of *CBS* and *CSE* overexpression on lifespan, stress resistance and locomotor activity.** ROS – reactive oxygen species. Please note that this scheme serve as generalized illustration and the mechanisms are dependent on the experimental system and model organism used. For detailed description of these mechanisms see [4–10].

Taken together, these data demonstrate that of *CBS* and *CSE* overexpression implicated in the control of stress resistance and aging process. The transgene overexpression of the *CSE* and *CBS* in *Drosophila* induce similar effects on stress-resistance and locomotor activity, however lifespan extending effect was revealed for *CBS* overexpression only. The positive effects of *CBS* and *CSE* overexpression on lifespan, stress resistance and locomotor activity may be associated with H_2_S-mediated modulation of autophagy, inflammation, oxidative stress, cell death, cellular metabolism and cellular bioenergetics (Figure 4). It should be taken into account that the effects of H_2_S are dependent on the experimental system, model organism, concentration, and may vary from beneficial to harmful [6–10].

## METHODS

### *Drosophila melanogaster* lines

Full length sequenced cDNA Clones from *Drosophila* Genomics Resource Center (DGRC, USA) were used for UAS lines creation. Cloning of cDNAs into *pUAST-attB* vector, sequence verification of each clone, and φC31 integrase-mediated site-specific germline transformation via embryo injection were ordered in GenetiVision (Houston, Texas, USA) (Table S1). *UAS-CSE[LD22661]* contains an additional copy of *CG12264* gene coding for mitochondria-located enzyme with CSE activity [18]. *UAS-CSE[LD22255]* carries an additional copy of *CG5345* gene that is the *Drosophila* orthologue of *CSE* (cytoplasm-located) [11]. *UAS-CBS[LD21426]* contains an additional copy of *CG1753* gene that is the single homolog of *CBS* in *Drosophila* [4].

The GAL4 line (GAL4 driver) was used for overexpression of UAS transgenes. *da-GAL4* expresses GAL4 ubiquitously and strongly under the control of *daughterless* [28]. This driver expresses throughout development and in most adult tissues [29]. *da-GAL4* line was kindly provided by Dr. Laurent Seroude (Queen’s University, Kingston, Canada).

In order to match the genetic background of UAS and GAL4 lines they were backcrossed into white-eyed standard *w^1118^* (#3605, Bloomington *Drosophila* Stock Center, USA) for 6-8 times.

### Activation of transgenes overexpression

The GAL4-UAS binary transgenic system was used to activate the expression of the studied genes [30]. We used constitutively active (*da-GAL4*) driver of GAL4 that activates the gene overexpression ubiquitously. The flies with overexpressed transgenes were obtained by mating the females from UAS lines and males from GAL4 driver lines.

### Quantitative real time PCR

To confirm overexpression of studied genes in the whole body 10-15 imagoes were used in every variant of the experiment. Gene expression levels were analyzed in flies at the age of 5-6 days after imago hatching. Experiments were performed in 3-4 replicates. Whole flies or heads were homogenized with the Silent Crusher-S homogenizer (Heidolph, Germany) in TRIzol Reagent (Invitrogen, USA). RNA was separated using BCP (Invitrogen, USA), in accordance with the manufacturer’s protocol. To test that RNA samples were DNA-free, control PCR experiments without the reverse transcription step were performed with primers for the *β-Tubulin* gene. Reverse transcription was performed using an Oligo(dT)_20_ primer (Invitrogen, USA) and SuperScript III Reverse Transcriptase (Invitrogen, USA), according to manufacturer’s instructions.

Quantitative real-time PCR (qRT-PCR) assays were performed using SYBRGreen PCR Master Mix (Applied Biosystems, USA). The list of primers is presented in Table S2. All reactions were performed using a CFX96 real-time PCR detection system (Bio-Rad Laboratories, USA). The thermal cycle conditions were: initial denaturation step at 95°С for 10 min, followed by 50 cycles of 95°С for 15 s (denaturation), 60°С for 30 s (annealing) and 60°С for 30 s (elongation). Expression levels were normalized against the housekeeping gene *β-Tubulin.* All target genes and *β-Tubulin* were amplified in separate PCR tubes. Four measurements were performed for each version of the experiment.

### Lifespan analysis

Control and experimental fly males were collected during 24 h after imago hatching and maintained in constant conditions on a sugar-yeast medium at 25°C and 60% humidity in a 12:12 h light-dark cycle. The flies were housed in *Drosophila* vials at a density of 30 individuals per vial, with 5 vials per experimental variant. Dead flies were recorded daily. Fresh medium vials were provided two times per week. Experiments were performed in 2 replicates. The median lifespan, the age of 90% mortality (maximum lifespan), and the mortality rate doubling time (MRDT) were calculated.

### Analysis of locomotor activity

Age-dependent changes in spontaneous locomotor activity were measured using the *Drosophila* Population Monitor (TriKinetics Inc., USA). Activity was defined as total number of sensor crosses during 3 min by 30 flies. Measurements were carried out every 10 days, starting from the age of 5 days, until 30 flies were alive in control and experimental variants (at the age of 5, 15 and 25 days).

### Stress resistance analysis

The resistance to acute stress conditions was evaluated in 5 days old imagoes. The exposure to paraquat (oxidative stress), hyperthermia, and combination of arid and food-free conditions were used. Before as exposed to paraquat flies were deprived of food and water for 3 h and then transferred into vials containing 3-layer filter paper saturated with 300 ml of a 5% sucrose solution with 20 mM paraquat (Sigma-Aldrich, USA). At the variant of hyperthermia, flies were kept in vials on agar-yeast medium at 35 °C. For combination of arid and food-free conditions flies were placed into empty vials. Flies were transferred into new vials every two days, and kept under stressful conditions until the death.

For evaluating the resistances to oxidative stress and heat shock the amount of dead flies were counted two times per day. For evaluating the resistances to combination of arid and food-free conditions dead flies were counted every 3 hours. The survival time for 25%, 50%, 75% and 90% of populations were estimated.

### Statistical analysis

Relative levels of expression were calculated using 2^-ΔΔСt^ method [31]. *ΔΔCt* was calculated according to equation *ΔΔCt = ΔCt (experiment) – ΔCt (control),* where *ΔCt = Ct (target gene) – Ct (β-Tubulin).* Statistical significance of expression differences was estimated using Mann-Whitney U-test [32]. To compare the statistical differences in survival functions and median lifespan between control and experimental groups, the modified Kolmogorov-Smirnov and log-rank [33] tests were used, respectively [34,35]. A Wang-Allison test was used to estimate the differences in the age of 90% mortality [36]. The χ^2^ test was used for locomotor activity and fecundity analysis. To assess the statistical significance of differences in resistance to stress factors, the Fisher's exact test was used [37]. Statistical analyses of the data were performed using R, version 2.15.1 (The R Foundation) and OASIS 2: Online Application for Survival Analysis 2 [37].

## Supplementary Material

Supplementary Figure

Supplementary Tables
