# Supplementary material for: Overexpression of CBS and CSE genes affects lifespan, stress resistance and locomotor activity in Drosophila melanogaster
Source: Aging (Albany NY). 2018 Nov 8;10(11):3260–72. doi: 10.18632/aging.101630 (PMC6286861; doi:10.18632/aging.101630)
Supplement: Supplementary Figure [file aging-10-101630-s001.pdf]

SUPPLEMENTARY FIGURE

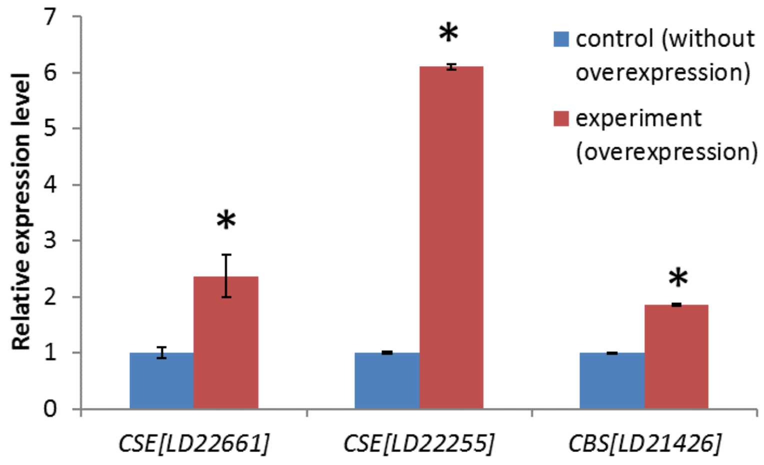

Figure S1. The relative level of expression of CSE and CBS genes under control of constitutive ubiquitous da-GAL4 driver. *$p<0.05$, Mann-Whitney U-test. Error bars indicate standard error of the mean.
