# Supplementary material for: Overexpression of CBS and CSE genes affects lifespan, stress resistance and locomotor activity in Drosophila melanogaster
Source: Aging (Albany NY). 2018 Nov 8;10(11):3260–72. doi: 10.18632/aging.101630 (PMC6286861; doi:10.18632/aging.101630)
Supplement: Supplementary Tables [file aging-10-101630-s002.pdf]

SUPPLEMENTARY TABLES

Table S1. Clones of cDNAs and docking sites of *pUAST-attB* vector in *UAS* lines.

UAS strains	Gene	DGRC Clone	Docking Site
UAS-CSE[LD22661]	*CSE/CG12264*	LD22661	2L: 22A3
UAS-CSE[LD22255]	*CSE/CG5345*	LD22255	3L: 62E1
UAS-CBS[LD21426]	*CBS/CG1753*	LD21426	2L: 22A3

Table S2. List of primers for qRT-PCR.

Gene	Forward	Reverse
CSE/CG12264	5'-CTTTCTTTGCGTGAGATGGA-3'	5'-GTATGTGGCTTTGGCATCAC-3'
CSE/CG5345	5'-GTGTGGATCGAGTCACCAAC-3'	5'-GGAGGTCAGGAAGGTGTTGT-3'
CBS/CG1753	5'-AGATTACGCCCAACATCCTC-3'	5'-CTCGCACTTGGCATACATCT-3'
β-Tubulin	5'-GGCCAACTGAACGCTGATCT -3'	5'-AAGCCGGGCATGAAGAAGTG-3'

Table S3. Relative expression level of CSE[LD22661], CSE[LD22255], CBS[LD21426] genes driven by da-GAL4.

Gene	Males		Females	
	Control	Overexpression	Control	Overexpression
CSE[LD22661]	1.00±0.09	2.37±0.37*	1.00±0.15	1.31±0.36
CSE[LD22255]	1.00±0.02	6.10±0.04*	1.00±0.01	3.75±0.04*
CBS[LD21426]	1.00±0.01	1.86±0.02*	1.00±0.02	1.06±0.03

*p<0.05, Mann-Whitney U-test. Errors indicate standard error of the mean.

Table S4. The P-values for differences in survival functions at specific time points (times of 25%, 50%, 75% and 90% mortality) calculated with Fisher's exact test.

Variants	Sex	Conditions	P-value at 25%	P-value at 50%	P-value at 75%	P-value at 90%
da-GAL4>UAS-CSE[LD22661] v.s. da-GAL4	males	paraquat	0.0428	0.0428	0.7828	0.7828
da-GAL4>UAS-CSE[LD22661] v.s. UAS-CSE[LD22661]	males	paraquat	0.0299	0.0299	0.6287	0.6287
da-GAL4>UAS-CSE[LD22255] v.s. da-GAL4	males	paraquat	0.1217	0.1217	0.0964	0.0964
da-GAL4>UAS-CSE[LD22255] v.s. UAS-CSE[LD22255]	males	paraquat	1	1	0.8279	1
da-GAL4>UAS-CBS[LD21426] v.s. da-GAL4	males	paraquat	0.2198	0.2198	0.7826	0.7826
da-GAL4>UAS-CBS[LD21426] v.s. UAS-CBS[LD21426]	males	paraquat	0.412	0.412	1	1
da-GAL4>UAS-CSE[LD22661] v.s. da-GAL4	males	hyperthermia	6.9e-13	4.8e-13	4.8e-13	1
da-GAL4>UAS-CSE[LD22661] v.s. UAS-CSE[LD22661]	males	hyperthermia	1.1e-12	4.8e-13	4.8e-13	1

da-GAL4>UAS-CSE[LD22255] v.s. da-GAL4	males	hyperthermia	6.2e-13	1.3e-12	0.0008	0.0008
da-GAL4>UAS-CSE[LD22255] v.s. UAS-CSE[LD22255]	males	hyperthermia	2e-13	0.0008	0.0008	0.0008
da-GAL4>UAS-CBS[LD21426] v.s. da-GAL4	males	hyperthermia	4.1e-13	0.0234	0.0234	0.0234
da-GAL4>UAS-CBS[LD21426] v.s. UAS-CBS[LD21426]	males	hyperthermia	6e-13	0.0234	0.0234	0.0234
da-GAL4>UAS-CSE[LD22661] v.s. da-GAL4	males	arid and food-free	8.6e-13	3.7e-13	1.2e-11	0.0154
da-GAL4>UAS-CSE[LD22661] v.s. UAS-CSE[LD22661]	males	arid and food-free	1.1e-12	1.1e-12	6.1e-7	1
da-GAL4>UAS-CSE[LD22255] v.s. da-GAL4	males	arid and food-free	7.6e-13	7.3e-13	1e-12	0.0051
da-GAL4>UAS-CSE[LD22255] v.s. UAS-CSE[LD22255]	males	arid and food-free	1.6e-12	6.7e-13	8.5e-13	1.1e-7
da-GAL4>UAS-CBS[LD21426] v.s. da-GAL4	males	arid and food-free	5.3e-12	1.1e-7	2.8e-12	0.0068
da-GAL4>UAS-CBS[LD21426] v.s. UAS-CBS[LD21426]	males	arid and food-free	0.0098	0.0258	0.0052	0.014
da-GAL4>UAS-CSE[LD22661] v.s. da-GAL4	females	paraquat	2.7e-9	0.0005	1	1
da-GAL4>UAS-CSE[LD22661] v.s. UAS-CSE[LD22661]	females	paraquat	6.8e-7	0.0009	1	1
da-GAL4>UAS-CSE[LD22255] v.s. da-GAL4	females	paraquat	9.3e-13	9.7e-9	1	1
da-GAL4>UAS-CSE[LD22255] v.s. UAS-CSE[LD22255]	females	paraquat	0.0000064	0.0000064	0.0005	1
da-GAL4>UAS-CBS[LD21426] v.s. da-GAL4	females	paraquat	1.5e-12	0.0000014	1	1
da-GAL4>UAS-CBS[LD21426] v.s. UAS-CBS[LD21426]	females	paraquat	0.869	0.869	0.1968	1
da-GAL4>UAS-CSE[LD22661] v.s. da-GAL4	females	hyperthermia	1.4e-12	1.5e-12	6.8e-13	1
da-GAL4>UAS-CSE[LD22661] v.s. UAS-CSE[LD22661]	females	hyperthermia	4.9e-13	4.8e-13	1.8e-12	1
da-GAL4>UAS-CSE[LD22255] v.s. da-GAL4	females	hyperthermia	5.7e-13	5.7e-13	3.3e-8	1
da-GAL4>UAS-CSE[LD22255] v.s. UAS-CSE[LD22255]	females	hyperthermia	0.0001	0.5088	1	1
da-GAL4>UAS-CBS[LD21426] v.s. da-GAL4	females	hyperthermia	1e-12	1e-12	9e-9	1
da-GAL4>UAS-CBS[LD21426] v.s. UAS-CBS[LD21426]	females	hyperthermia	7.9e-9	8.1e-9	8.1e-9	1
da-GAL4>UAS-CSE[LD22661] v.s. da-GAL4	females	arid and food-free	8e-13	6.7e-13	6.7e-13	0.0318
da-GAL4>UAS-CSE[LD22661] v.s. UAS-CSE[LD22661]	females	arid and food-free	1.2e-11	5.6e-13	8.9e-13	0.031
da-GAL4>UAS-CSE[LD22255] v.s. da-GAL4	females	arid and food-free	5.6e-13	9.7e-13	3e-13	0.0000015
da-GAL4>UAS-CSE[LD22255] v.s. UAS-CSE[LD22255]	females	arid and food-free	1.1e-12	4.1e-13	3.3e-10	0.0000071
da-GAL4>UAS-CBS[LD21426] v.s. da-GAL4	females	arid and food-free	2.4e-10	0.000029	0.0000015	0.0000015
da-GAL4>UAS-CBS[LD21426] v.s. UAS-CBS[LD21426]	females	arid and food-free	0.1506	0.0443	0.0443	0.0032
